# *Bacillus amyloliquefaciens* YN201732 Produces Lipopeptides With Promising Biocontrol Activity Against Fungal Pathogen *Erysiphe cichoracearum*

**DOI:** 10.3389/fcimb.2021.598999

**Published:** 2021-06-18

**Authors:** Rong Jiao, Yongzhan Cai, Pengfei He, Shahzad Munir, Xingyu Li, Yixin Wu, Junwei Wang, Mengyuan Xia, Pengbo He, Ge Wang, Huanwen Yang, Samantha C. Karunarathna, Yan Xie, Yueqiu He

**Affiliations:** ^1^ State Key Laboratory for Conservation and Utilization of Bio-resources in Yunnan, Yunnan Agricultural University, Kunming, China; ^2^ Science and Technology Division, Yuxi Normal University, Yuxi, China; ^3^ Qujing Tobacco Co., Ltd., Qujing, China; ^4^ Hongta Tobacco (Group) Co., Ltd., Yuxi, China; ^5^ Faculty of Tobacco Science, Yunnan Agricultural University, Kunming, China; ^6^ CAS Key Laboratory for Plant Diversity and Biogeography of East Asia, Kunming Institute of Botany, Chinese Academy of Science, Kunming, China

**Keywords:** antifungal activity, bacillomycin, biofilm, tobacco powdery mildew, pathogen

## Abstract

*Bacillus amyloliquefaciens* YN201732 is an endophytic bacteria with high biocontrol efficiency and broad-spectrum antimicrobial activities. In order to clarify the main active ingredients and their antifungal mechanisms against powdery mildew of tobacco, this study is focused on lipopeptide obtained through acid precipitation and organic solvent extraction. HPLC and LCMS-IT-TOF were used to separate and identify antimicrobial lipopeptides. Findings revealed that bacillomycin D plays an important role against surrogate fungal pathogen *Fusarium solani*. Synthetic pathways of sfp, bacillomycin D, and fengycin were separately disrupted. The *sfp* gene knockout mutant *B. amyloliquefaciens* YN201732M1 only showed minor antagonistic activity against *F. solani*. While *Erysiphe cichoracearum* spore germination was inhibited and pot experiments displayed a significant decrease in tobacco powdery mildew. The spore inhibition rate of YN201732M1 was only 30.29%, and the pot experiment control effect was less than 37.39%, which was significantly lower than that of the wild type. The inhibitory effect of mutant YN201732M2 (deficient in the production of bacillomycin D) and mutant YN201732M3 (deficient in the production of fengycin) on the spore germination of *E. cichoracearum* were 50.22% and 53.06%, respectively, suggesting that both fengycin and bacillomycin D had potential effects on spore germination of powdery mildew. Interestingly, in a greenhouse assay, both *B. amyloliquefaciens* YN201732M2 and YN201732M3 mutants displayed less of a control effect on tobacco powdery mildew than wild type. The results from *in vitro*, spore germination, and greenhouse-pot studies demonstrated that antimicrobial lipopeptides especially bacillomycin D and fengycin may contribute to the prevention and control of tobacco powdery mildew. In addition, gene mutation related to lipopeptide synthesis can also affect the biofilm formation of strains.

## Introduction

*Erysiphe cichoracearum* is the primary cause of powdery mildew in tobacco plants, and it results in substantial reduction of yields and quality of tobacco in China (Xing et al., 2015). This pathogen is a biotrophic parasite, which is harmful to mature leaves and spreads from the bottom to the top of leaves (Dietz and Winter, 2019). Disease symptoms begin with the development of white powdery disease spots on both sides of leaves, and the spots expand followed by foliar chlorosis with pathogen spread all over the leaves, eventually leading to plant death (Glawe, 2008). The application of chemical fungicides is the main strategy for controlling tobacco powdery mildew. However, with increasing concern over fungicide resistance, environment pollution, and food safety (Koch et al., 2018; Patle et al., 2018), biological control has attracted considerable attention.

*Bacillus* as an ideal biocontrol bacterium has been used for biological control of crop diseases and insect pests, especially endophytic *B. amyloliquefaciens*, which are ubiquitously found to be safe microorganisms with proven excellent *in planta* colonization aptitudes (Cao et al., 2011; Liu et al., 2017) and versatility in effectively protecting plants from pathogens (Ongena and Jacques, 2008). They can compete with pathogens for nutrients, secretes antimicrobial substances, and induce the plant’s defense system to resist the invasion of pathogens (Wang et al., 2017). The most direct way through which *B. amyloliquefaciens* suppresses pathogens is the production of secondary metabolites such as antimicrobial proteins, lipopeptides, polyenes, phospholipids, amino acids, nucleic acids, and polyketides (Ongena and Jacques, 2008; Chen et al., 2015; Yan et al., 2018). Low-molecular-weight lipopeptides are one of the most common types of antimicrobial substances (Koumoutsi et al., 2004). Iturin, fengycin, and surfactin are the three most studied families of cyclic peptides (LPs). With a unique chemical composition and amphiphilic ring structure, they can still maintain high activity under high temperature, ultraviolet rays, and different pH values and can also resist the hydrolysis action of peptidases and proteases (Santoyo et al., 2012; Caulier et al., 2019). The iturin and fengycin families are associated with strong antifungal activity against various fungi *in vitro*, while the surfactant family is responsible for less antifungal activity, but displays the properties of a surfactant (Romero et al., 2007). Guo et al. (2019) reported that *B. subtilis* NCD-2 inhibits pathogenic fungi by producing fengycin-type cyclic peptides, which may play a role in the suppression of clubroot pathogen on Chinese cabbage. Since the discovery of secondary metabolites secreted by *Bacillus* to inhibit or even kill plant pathogens (Stein, 2005), scientists have been working on the application of microbial antagonists to manage plant diseases (Li et al., 2018; Munir et al., 2020). Microbial communities also play a significant role to manage the bacterial wilt of tobacco (Cai et al., 2021). Studies have found that lipopeptides produced by *B. amyloliquefaciens* FZB42 are necessary for inducing resistance, biofilm formation, root colonization, and successful control of plant pathogens (Koumoutsi et al., 2004; Chowdhury et al., 2015). In addition, antimicrobial peptides are powerful weapons of biocontrol strains against other pathogenic microorganisms, and their various structural types can help *Bacillus* effectively avoid the development of pathogen resistance and thus maintain the antagonistic advantage of biocontrol agents (Raaijmakers et al., 2010).

*Bacillus amyloliquefaciens* YN201732 is a beneficial endophytic strain isolated from tobacco seeds that can suppress the growth of *E. cichoracearum* in tobacco and protect the host from pathogen invasion through efficient root colonization (Jiao et al., 2020). *In vitro* testing showed that strain YN201732 can effectively antagonize a variety of pathogenic fungi, and the supernatant of *B. amyloliquefaciens* YN201732 completely inhibited the conidial germination of *E. cichoracearum*, implying that it may secrete antifungal compounds (Jiao et al., 2020). In this study, the antifungal LPs were investigated using both biochemical and genetic approaches. The crude extract of lipopeptides in *in vitro* tests and gene disruption experiments confirmed that bacillomycin D and fengycin were the major compounds among the detected LPs, responsible for the inhibition of *E. cichoracearum* in tobacco. Further in-depth research on the main antimicrobial substance of *B. amyloliquefaciens* YN201732 against phytopathogen is of great significance for the development of endophyte YN201732 as biological pesticides and bio-organic fertilizers.

## Materials and Methods

### Microorganisms and Plasmids

The strains and plasmids used in this study are described in **Table 1**. *Escherichia coli* TG1 competent cells were used as a host for all plasmids. *B. amyloliquefaciens* strain YN201732 was identified following the method described in Jiao et al. (2018) and stored in 50% glycerol at -80°C (in Laboratory of Biocontrol and Plant Pathology, Yunnan Agricultural University, Kunming, China). Since *E. cichoracearum* cannot be cultured purely, the pathogen was collected from the surface of naturally infected leaves. Pathogen *F. solani* preserved by the Laboratory of Biocontrol and Plant Pathology, Yunnan Agricultural University, Kunming, China, and maintained on potato dextrose agar (PDA) medium, was used as an indicator fungus to overcome the problem of *in vitro* culturing of powdery mildew pathogen.

**Table 1:** Microorganism and plasmid used in this study.

Plasmid/Strain	Characteristics	Source or reference
**Fungus**		
*Fusarium solani*	Pathogen of *Panax notoginseng* root rot	Laboratory stock
*Erysiphe cichoracearum*	Pathogen of tobacco powdery mildew	Natural infected
**Bacteria**		
*Escherichia coli* TG1	*supE hsdΔ5 thi Δ(lac-proAB)*/F’ [*traD36proAB^+^ lacI^q^lacZ ΔM15*]	Laboratory stock
*B. amyloliquefaciens* YN201732	Wild type	
*B. amyloliquefaciens* YN201732M1	*B. amyloliquefaciens* YN201732Δsfp:: Kan	This study
*B. amyloliquefaciens* YN201732M2	*B. amyloliquefaciens* YN201732ΔbmyA:: KanR	This study
*B. amyloliquefaciens* YN201732M3	*B. amyloliquefaciens* YN201732ΔfenB:: KanR	This study
**Plasmid**		
pUC18	Amp^r+^, 2.7kb, ori from pBR322	TaKaRa (Dalian)
pMD18-T	Amp^r+^, 2.7kb, linear pUC18 with T overhangs	TaKaRa (Dalian)
pBluscript KS minus	Amp^r+^, 3.0kb, ori from pBR322	Laboratory stock
pMD18-kanR	Amp^r+^, Kan^r+^, pMD18 insert with KanR, 3.7kb	Laboratory stock
pBEST502	Amp^r+^, Kan^r+^, ori from pBR322	(Itaya and Tanaka,1990)

### Preparation of Crude Extracts of Lipopeptide Compounds and Separation and Purification of Organic Solvents

For lipopeptide production, liquid chromatography and mass spectrometry characterization were used. *Bacillus amyloliquefaciens* YN201732 was streaked on Luria-bertani (LB) plates and cultured at 37°C for 24 hours. After growing, a single colony was picked and inoculated into Landy liquid medium at 37°C, 160 rpm, cultured for 48 hours with shaking to prepare 2 L of fermentation broth (Branda et al., 2001). The fermented suspension was centrifuged to remove bacterial cells (50 mL centrifuge tube, 4°C, 10000 rpm, 20 minutes), and the supernatant was adjusted to pH 2.0 with 6 mol/L HCl and left at 4°C overnight. The precipitate was collected by centrifugation at 10,000 rpm for 10 minutes. The precipitate was further washed three times with sterile deionized water, 5 mL of deionized water was also added to adjust pH to 7.0 with 1 mol/L NaOH. After freeze-drying, the powder was extracted with three to four times the amount of 95% methanol under the assistance of ultrasonic for 3 hours. After centrifugation, the precipitate was collected and extracted twice, and the extracted solution was mixed together. The pooled extraction solution was completely evaporated under pressure using a rotary vacuum evaporator to obtain a crude extract of the lipopeptide. Then the lipopeptide crude extract was sequentially extracted with petroleum ether, ethyl acetate, n-butanol, and water. After the crude extract was dissolved in 100 mL H_2_O, it was extracted with 1: 1 (V: V) petroleum ether for 3 hours. During this period, it was shaken every 10 minutes and extracted 3 times. Organic layer combined extracts were concentrated and weighed with a rotary evaporator. Aqueous phase was sequentially extracted with ethyl acetate and n-butanol in the same way, and the remaining aqueous phase was directly concentrated and weighed to obtain secondary extracts of four different polar solvents. The four separated components were concentrated and dried and then dissolved in a certain amount of methanol. The solvents were volatilized and secondary extraction product was dissolved with equal amounts of methanol respectively, and four extracted fractions were collected. Four fractions were filtered through a 0.22-μM-pore-size hydrophilic membrane, which was used for the determination of antifungal activity.

### Determination of Antifungal Activity of Various Components of Crude Lipopeptides Against Pathogenic Fungi

The phytopathogenic fungus *F. solani* was cultured on the PDA for 3 days, and the hyphal pieces were picked from the edge of the colony, and inoculated into a 250 mL flask containing 100 mL of a sterilized PDA broth at 25°C, with 160 rpm for 3 to 5 days. Large mycelium was removed by filtering through sterile gauze to obtain a conidial suspension, counted by using a blood cell counter, and the concentration was adjusted to 10^8^ cfu/mL with sterile water. One hundred microliters (100 μL) of the conidia suspension of *F. solani* was spread on PDA plates. A volume of 100 μL of the collected fraction was dropped into the hole (5mm) 2.5 cm from the center of the Petri plate and allowed to diffuse into the agar. Plates were then incubated at 25°C, and the distance between the edges of the Petri dish and the fungal mycelium was measured after 5 days. The solvent methanol was set as the negative control, and the antimicrobial lipopeptide methanol crude extraction was the positive control at the same time. The experiment was repeated three times.

### LCMS-IT-TOF Analysis of Lipopeptide Active Components

Liquid chromatography conditions: qualitative analysis of active LPs were performed using high-performance liquid chromatography (HPLC) (Agilent 1200, U.S.). A sample of the active secondary extract methanol solution was taken, filtered through a 0.22 μM microporous filter, and then loaded. The injection was 5 μL, and the column was LH-20 (MeOH) (Welch Ultimate AQ-C18, 5 μM, φ 4.6 × 300 mm). The column temperature, detection wavelength, and flow rate were 35°C, 254 nm, and 1 mL/min, respectively. The active part of the mobile phase was acetonitrile: the water (containing 0.05% trifluoroacetic acid) (v/v) was 40:60.

Mass spectrometry (MS) conditions: the high-performance liquid ion trap time-of-flight mass spectrometer was LCMS-IT-TOF (Shimadzu Corporation, Japan). LCMS-IT-TOF is an emerging spectrometry technique that is a combination of both QIT (ion trap) and TOF (time-of-flight). An accurate mass-to-charge ratio of lipopeptides can be obtained without complicated extraction, separation, and purification steps, and the types of lipopeptides can be qualitatively judged by comparing with the molecular weight of known compounds. In the ESI ion source, the detection method was positive and negative ions, and the atomizing gas flow rate was 1.50 L/min. While the temperature of the curve desolvent tube (CDL) and the heating module was 200°C, the PR vacuum degrees of the IT vacuum and TOF vacuum were 97 Pa, 1.1 × 10^-2^ Pa, and 1.2 × 10^-4^ Pa, respectively. Collision-induced dissociation technology (CID) was used to obtain the typical fragment ions of the compound. The detector voltage was 1.50 kv. High-purity argon was used as collision gas in the collision cell with a collision energy of 20–50%. Accurate mass analysis was performed on the main chromatographic peaks, and chemical composition was confirmed by comparison with related literature.

### Inhibitory Effect of Lipopeptide Gene Mutation on *Fusarium solani*

In order to further verify the role of lipopeptide antibiotics produced by *B. amyloliquefaciens* YN201732 to control disease, a double-exchange homologous recombination method (Liu et al., 2018) was used to knock out the 4’-phosphopantetheinyl transferase gene (*sfp*), non-ribosomal pathway peptide antibiotics bacillomycin D (*bmyA*) and fengycin synthetic genes (*fenB*). A 100 μL cell-free culture filtrate of *B. amyloliquefaciens* YN201732 and its lipopeptide mutants (incubated at 37° C for 48 hours in Landy medium), was loaded into 5 mm small wells from which agar pieces were taken out, and a plug (about 5 mm in diameter) of *F. solani* from a 5-day-old (25°C) PDA plate was placed in the center. Antagonistic activity of YN201732 and its mutants against *F. solani* was studied in the way of co-culture on the plate (Xu et al., 2013).

### Qualitative Biofilm Determination of *Bacillus amyloliquefaciens* YN201732 and Its Mutants

A biofilm assay was carried out using a modified version of the microtiter plate as described by Liu (Liu et al., 2018). The overnight-grown YN201732 and mutant were inoculated into fresh LB culture medium, and cultured at 37°C, 160 rpm to a bacterial solution concentration of OD600 of 1.0, making them in the logarithmic growth phase. The bacterial solution was centrifuged at 4°C and 10,000 rpm for 5 minutes, and bacterial cells were collected, washed three times with 0.9% NaCl solution, and resuspended in an equal volume of biofilm growth medium (MSgg medium, 5 mM potassium phosphate(pH 7), 100 mM morpholino propanesulfonic acid (pH 7), 0.5% glutamate, 50 μg/mL tryptophan, 50 μg/mL phenylalanine, 2 mM MgCl_2_, 700 μM CaCl_2_, 50 μM MnCl_2_, 50 μM FeCl_3_, 1 μM ZnCl_2_, 2 μM thiamine, 0.5% glycerol) (Branda et al., 2001). Qualitative biofilm determination was performed in 24-well polyvinylchloride (PVC) microtiter plates. In total, 2 mL of Msgg culture medium was added to the culture wells, and then 10 μL each of YN201732 wild-type and YN201732M1, YN201732M2, and YN201732M3 were set up in four replicates. The microtiter plates were incubated under stationary conditions at 37°C, and the film formation of YN201732 wild-type and mutant strains was observed after 12h and 24h, respectively.

### Inhibition Test of *Bacillus amyloliquefaciens* YN201732 and Its Mutants Against Spore Germination of Tobacco Powdery Mildew

The cell-free culture filtrates of *B. amyloliquefaciens* YN201732 and its mutants were mixed with 5% (w/v) water agar medium (Agar,5 g; ddH_2_O, 100 mL) in proportion to make a fermentation filtrate content of 20% (v/v) culture medium, invert a plate with a thickness of about 2 mm, and use a water agar plate containing 20% (v/v) sterile LB medium as a control. Naturally-occurring powdery mildew diseased leaves were shaken to evenly spread the spores on the plate and incubated at 25°C for 24 h. Moreover, after germination, 50 mm of the sterile puncher was used to create holes from the water agar medium which was mixed with the cell-free culture filtrates of YN201732 and its mutants, an agar plug was put on the center of a glass slide. The conidia were examined using a light microscope, and the germination standard was based on the length of the germ tube exceeding half the diameter (short diameter) of the spores. The experiment was repeated three times with each treatment repeated three times with five replicates. Microspores (150) were randomly examined through a microscope. The ratio of germinated spores to total observed spores was germination rate, and the inhibition rate of strain fermentation filtrate (%) = [Control spore germination rate – Treatment spore germination rate)/Control spore germination rate] × 100.

### Control Effect of *Bacillus amyloliquefaciens* YN201732 and Its Mutants on Powdery Mildew in Greenhouse

The experiment was carried out in the greenhouse of the College of Plant Protection, Yunnan Agricultural University, Kunming, China. Tobacco seedlings of Yunyan 87 (4–5-leaf-stage) were transplanted into a pot (10 cm × 12 cm) containing 200 g of autoclaved soil, 1 plant/pot. YN201732 wild type and its mutant strains were incubated in Landy medium at 37°C for 48 hours, and the cell concentration of YN201732 and its mutants was adjusted to 1 × 10^7^ cfu/mL. The disease base was investigated with the appearance of symptoms; sprayed the leaves with YN201732 wild type and its mutants suspension once, 10 mL/plant. Sterile LB medium and azoxystrobin 50% WG 10,000 times were used as negative and positive controls. Tween 20 (0.1%) was mixed with each agent prior to spraying. Initially, the disease symptoms were recorded after 3rd day and finally, with the interval of 7 days after inoculation of YN201732 wild type and its mutants. Disease severity was evaluated based on the percentage of leaves covered by mycelia and the percentage of powdery mildew caused by *E. cichoracearum* (tobacco pest index and survey standard, 2008), and the disease index and relative control effect were calculated. This experiment was performed in 3 replicates per treatment with 20 seedlings per replicate. The whole experiment was set according to a completely randomized block design.

### Statistical Analysis

The data were subjected to analysis of variance using IBM SPSS Statistics 22.0. The mean values among the treatment groups were compared using Duncan’s multiple range test at the 5% level of significance (*P* < 0.05).

## Results

### Antifungal Activity of Various Components of Crude Lipopeptides Against Pathogenic Fungi

**Figure 1:**
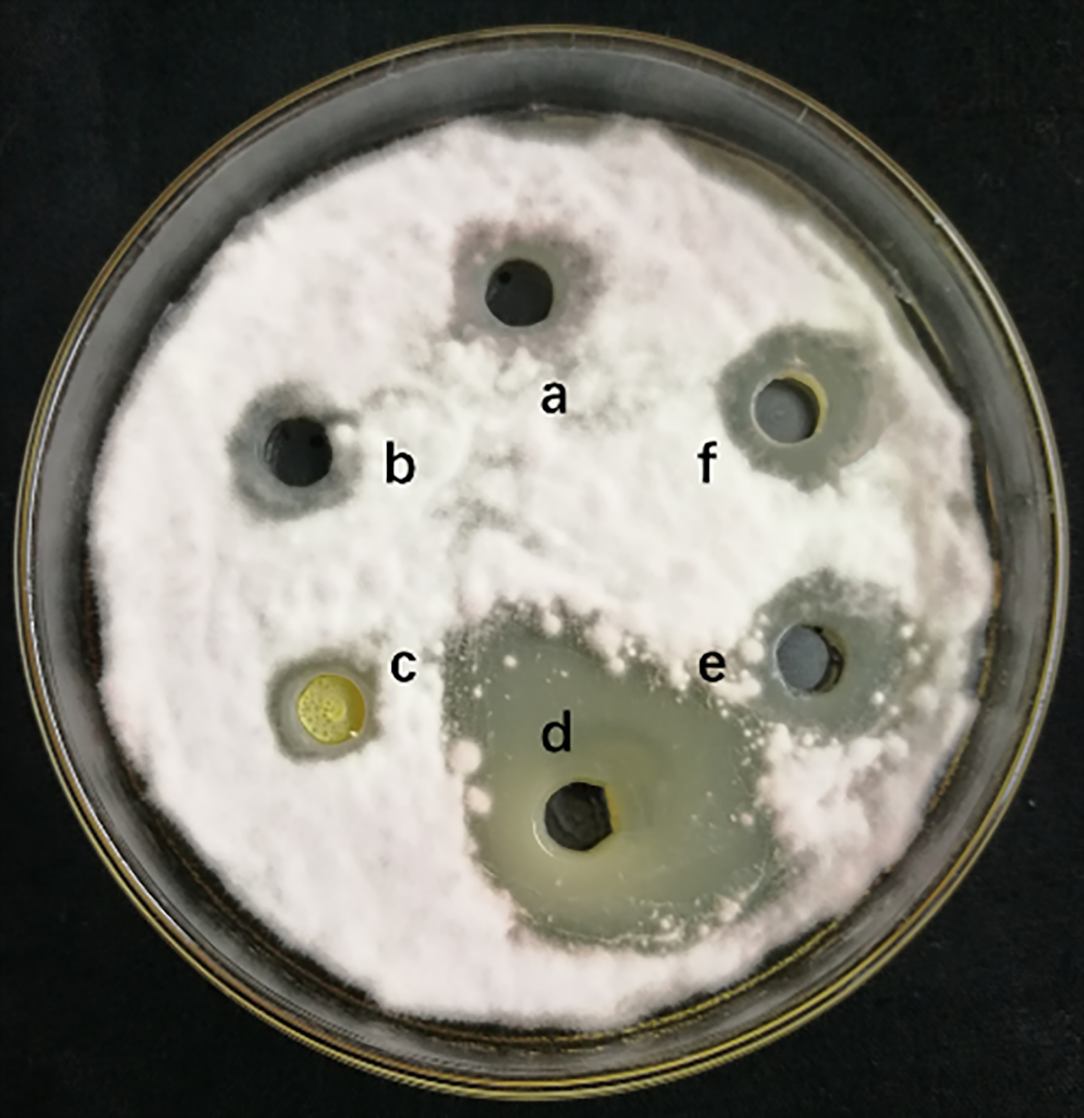
Inhibitory effect of different lipopeptides crude extract on *F. solani.* a-methanol (negative control), b-Petroleum ether extraction component, c- Ethyl acetate extraction component, d-N-butanol extraction component, e-Water extraction component, f- Methanol crude extract(positively control). A volume of 100 μL of the collected fraction was dropped into the hole on a PDA agar plate with actively growing *F. solani*. The plates were incubated for 5 days at 25°C.

The measurement of 4 different lipopeptides crude extract antagonistic activity is shown in **Figure 1**. The results show that the n-butanol extract had obvious antifungal activity against *F. solani*, and the average zone of inhibition diameter was 2.63 ± 0.17 cm.

### LCMS-IT-TOF Analysis of Active Substances in Lipopeptide Extracts

**Figure 2:**
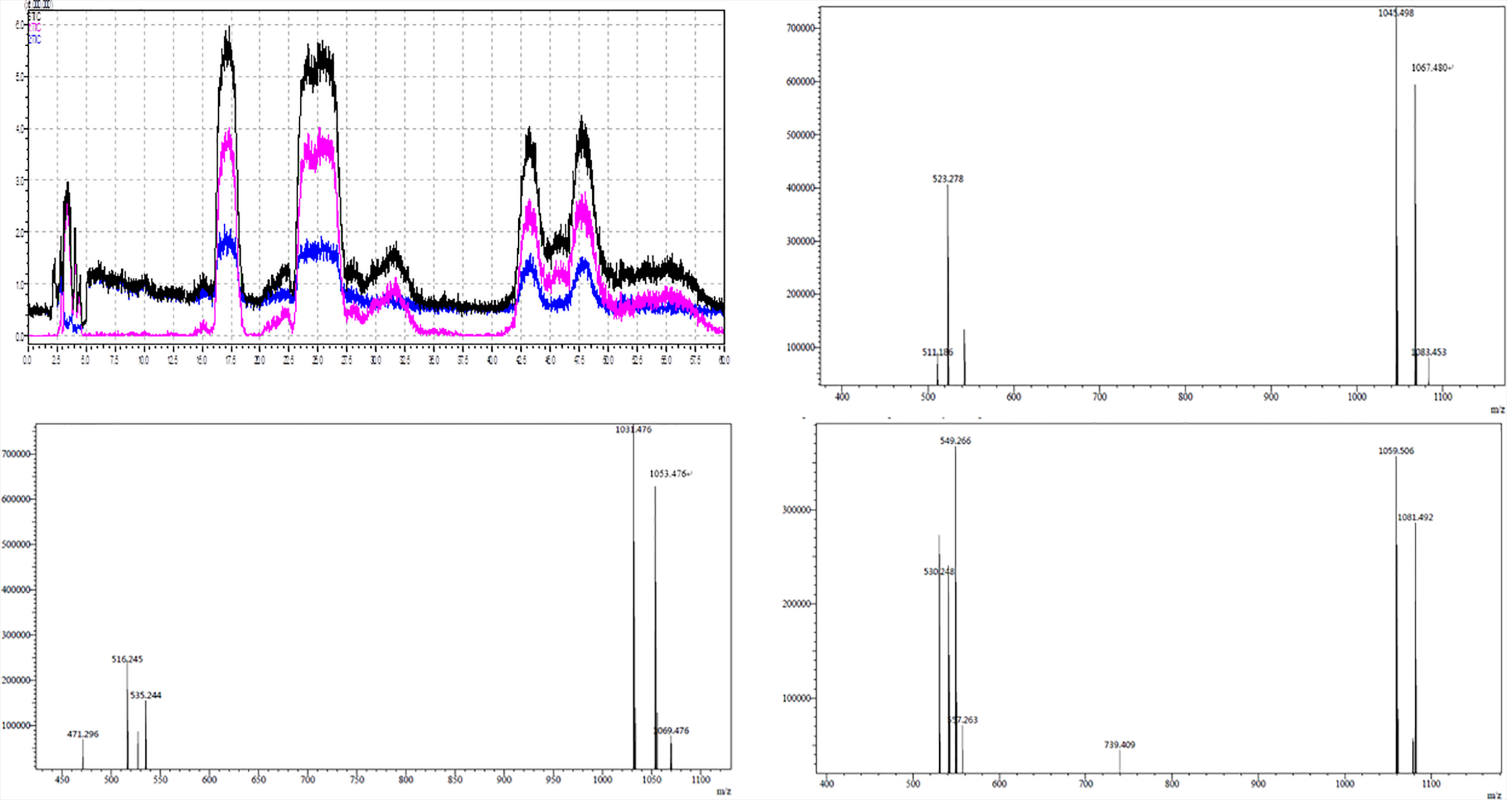
LCMS-IT-TOF of secondary metabolite produced by *B. amyloliquefaciens* YN201732.

The active components of the n-butanol extract were first optimized by HPLC and then detected by LCMS-IT-TOF mass spectrometer. Eight molecular ion peaks were detected for the corresponding seven retention time components (**Figure 2**) (m/z 1031.4, 1053.4, 1069.4, 1045.4, 1067.4, 1083.4, 1059.5, and 1081.4). By consulting relevant literature, comparing the molecular weights of lipopeptides of *Bacillus* (Yan et al., 2015; Caulier et al., 2019), it was found (see **Table 2**) that the mass-to-charge ratios m/z 1031.4, 1053.4, and 1069.4 correspond to the hydrogen ion peaks [M + H]^+^, the sodium ion peak [M + Na]^+^ and the potassium ion peak [M + K]^+^, whose corresponding molecular weights are both 1030.4. This compound belongs to the *Bacillus* antimycin D of the subtilisin family. The corresponding compound is 14-carbon C14-Bacillomycin D; mass-to-charge ratios m/z 1045.4, 1067.4, and 1083.4 correspond to the hydrogen ion peaks [M + H]^+^, the sodium ion peak [M + Na]^+^ and the potassium ion peak [M + K]^+^. The corresponding molecular weight is 1044.4, and the corresponding compound is C15-Bacillomycin D of 15 carbons; the mass-to-charge ratios m/z 1059.5 and 1081.4 correspond to the hydrogen ion peak [M + H]^+^ and the sodium ion peak [M + Na]^+^, its corresponding molecular weight is 1058.4 and the corresponding compound is C16-Bacillomycin D of 16 carbons. The same compound has different ionic forms, corresponding to three kinds of compounds, respectively. According to the above analysis, it can be found that the molecular weights of the three compounds differ in order by 14 Da, that is, one methylene group (-CH_2_), so these three compounds should be a group of homologs.

**Table 2:** Mass peaks of secondary metabolite compounds in n-butanol extract from *Bacillus amyloliquefaciens* YN201732.

No.	Retention time	Mass-to-charge ratio	Molecular weight	Positive ions	Compound
1	18.303	1031.4	1030.4	[M+H]^+^	C14-Bacillomycin D
2	18.303	1053.4	1030.4	[M+Na]^+^	C14-Bacillomycin D
3	18.303	1069.4	1030.4	[M+K]^+^	C14-Bacillomycin D
4	18.525	1031.4	1030.4	[M+H]^+^	C14-Bacillomycin D
5	18.525	1053.4	1030.4	[M+Na]^+^	C14-Bacillomycin D
6	18.525	1069.4	1030.4	[M+K]^+^	C14-Bacillomycin D
7	25.555	1045.4	1044.4	[M+H]^+^	C15-Bacillomycin D
8	25.555	1067.4	1044.4	[M+Na]^+^	C15-Bacillomycin D
9	25.555	1083.4	1044.4	[M+K]^+^	C15-Bacillomycin D
10	25.998	1045.4	1044.4	[M+H]^+^	C15-Bacillomycin D
11	25.998	1067.4	1044.4	[M+Na]^+^	C15-Bacillomycin D
12	25.998	1083.4	1044.4	[M+K]^+^	C15-Bacillomycin D
13	27.328	1045.4	1044.4	[M+H]^+^	C15-Bacillomycin D
14	27.328	1067.4	1044.4	[M+Na]^+^	C15-Bacillomycin D
15	27.328	1083.4	1044.4	[M+K]^+^	C15-Bacillomycin D
16	45.182	1059.4	1058.4	[M+H]^+^	C16-Bacillomycin D
17	45.182	1081.4	1058.4	[M+Na]^+^	C16-Bacillomycin D
18	49.837	1059.5	1058.5	[M+H]^+^	C16-Bacillomycin D
19	49.837	1081.4	1058.4	[M+Na]^+^	C16-Bacillomycin D

As shown in **Table 3**, m/z 1031.4 was detected in addition to the retention time RT 18.303, and was detected once in other time periods RT 18.525, indicating that m/z 1031.4 corresponds to two isomers. This phenomenon also exists in the other seven molecular ion peaks, which are m/z 1053.4 and 1069.4, which can also be detected at two different retention times of RT 18.303 and 18.525 min; m/z 1045.4, 1067.4, and 1083.4 at Rt 25.555, 25.998, 27.329 min were detected at three different retention times; m/z 1059.4, 1081.4 were detected at RT 45.182, 49.837 min. Since m/z 1031.4, 1053.4, and 1069.4 correspond to the same compound C14-Bacillomycin D, it is speculated that C14-Bacillomycin D has two isomers; m/z 1045.4, 1067.4, and 1083.4 correspond to the same compound C15-Bacillomycin D, and it is speculated that C15 -Bacillomycin D has three isomers; m/z 1059.4 and 1081.4 correspond to the same compound C16-Bacillomycin D. It can be considered that C16-Bacillomycin D has two isomers. However, the specific structural assignment of each substance cannot be judged based on the results of LCMS-IT-TOF, and further research is needed.

**Table 3:** Inhibitory effect of *Bacillus amyloliquefaciens* YN201732 and the mutants fermentation filtrate on the conidia germination of *Erysiphe cichoracearum*.

Treatment	Conidia germination rate/%	Inhibition rate/%
CK	25.54 ± 9.01^d^	
YN201732M1	17.80 ± 7.64^c^	30.29 ± 7.95
YN201732M2	12.71 ± 4.38^b^	50.22 ± 5.26
YN201732M3	11.99 ± 10.19^ab^	53.06 ± 9.04
YN201732	7.06 ± 4.75^a^	72.37 ± 3.15

Different letters indicate that the differences are significant (P < 0.05) using Duncan’s multiple range test.

### Inhibitory Effect of Microbial Lipopeptide Gene Mutants on *Fusarium solani*

**Figure 3:**
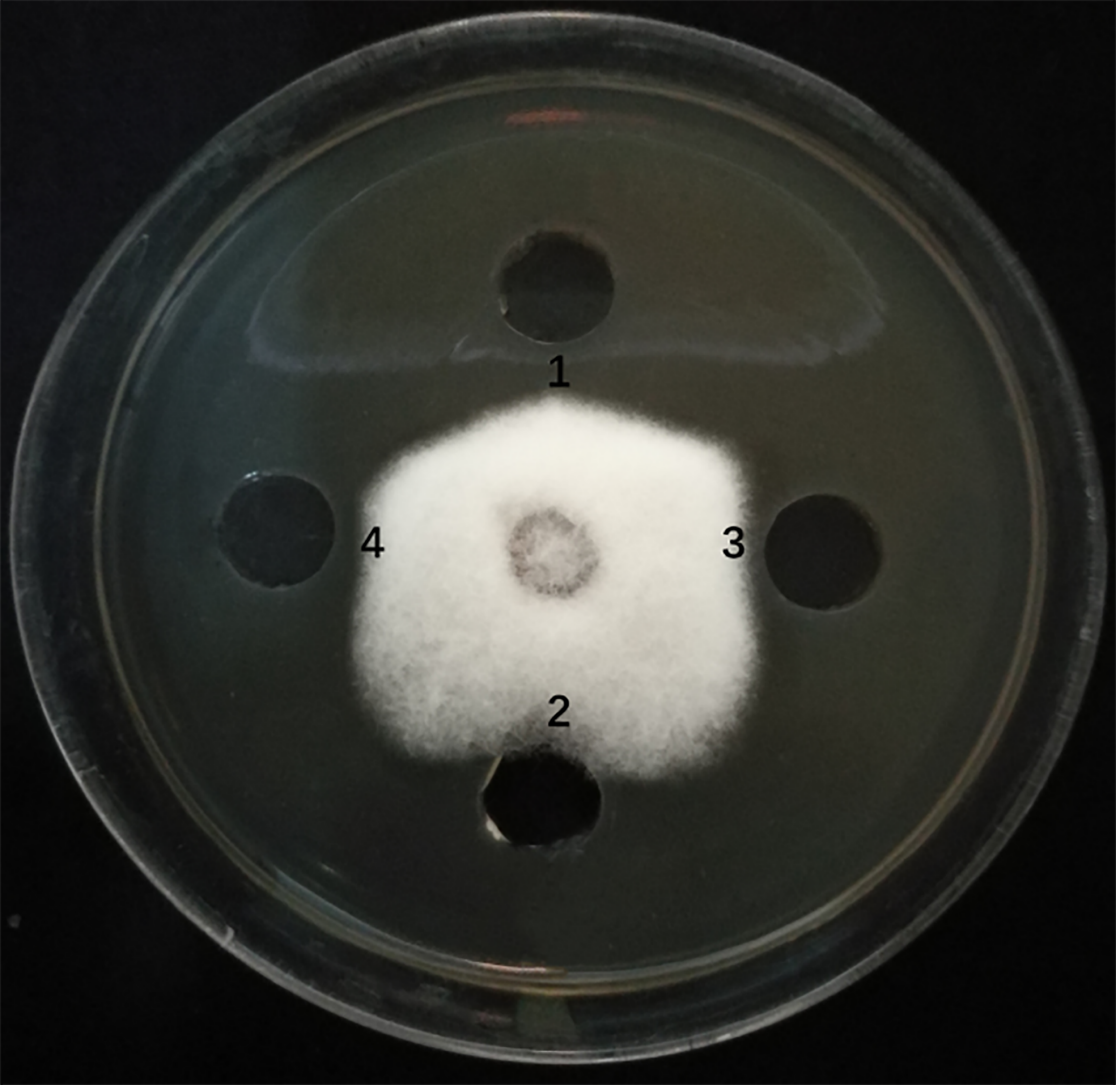
Antagonistic effects of supernatant drawn from *B. amyloliquefaciens* wild type strain YN201732 (spot 1), the sfp-disrupted mutant strain YN201732M1 (spot 2), the bmyA-disrupted mutant strain YN201732M2 (spot 3), and the fenB-disrupted mutant strain YN201732M3 (spot 4) against the indicator organisms (*F. solani*). A volume of 100 μL of a 48h culture grown in Landy medium was dropped into the hole (5mm) on PDA agar plates with actively growing *F. solani*. The plates were incubated at 25°C for 5 days.

The *sfp*, *bmyA*, and *fenB* genes in endophyte YN201732 were knocked out, and mutant strains YN201732M1, YN201732M2, and YN201732M3 were obtained, respectively. In order to study the effect of mutant strains on the antagonistic activity of *E. cichoracearum*, *F. solani* was used as indicator organisms, the supernatants of YN201732 wild-type and its mutant strains were used to perform a plate-to-pan test of *F. solani*. The results in **Figure 3** show that the supernatant of wild-type strain YN201732 inhibited the mycelial growth of *F. solani*, and the average distance between the edges of the petri dish and the fungal mycelium was 5.37 ± 0.46 mm, while the disruption of *sfp* had almost no effect on the antifungal activity against *F. solani*. Under the same condition, the supernatant of YN201732M2 showed minor antifungal activity against *F. solani*, with the average distance between the edges of the Petri dish and the mycelium being about 1.48 ± 0.46 mm. The inhibition zone of mutant YN201732M3 was 3.97 ± 0.06 mm, and the antifungal effect was slightly lower than that of the wild-type strain. These results show that antimicrobial lipopeptides except for fengycin could inhibit the pathogen of *F. solani*. These data confirmed the hypothesis that the LP, especially the fraction of bacillomycin D, plays a major role in *F. solani* inhibition.

### Comparison of *Bacillus amyloliquefaciens* YN201732 and Its Mutant Biofilm Formation Capabilities

**Figure 4:**
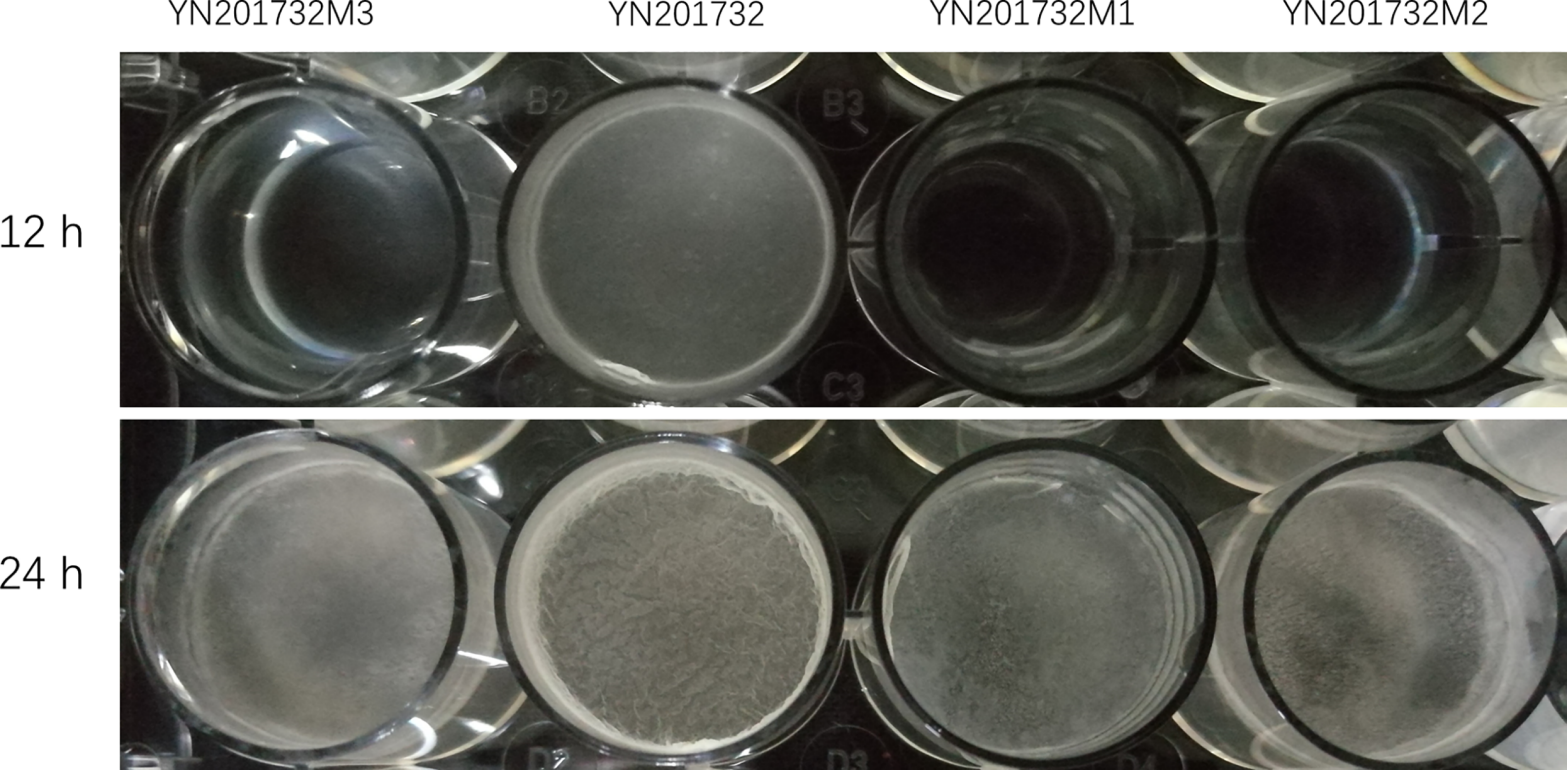
Microtiter plate assay of biofilm formation by wild type and the mutant strains. Wild-type YN201732, YN201732M1, YN201732M2, and YN201732M3 cells were grown in MSgg medium at 37°C for the indicated times.

The biofilms of wild-type YN201732 and mutants YN201732M1, YN201732M2, and YN201732M3 were grown in MSgg medium for 12h and 24h, as shown in **Figure 4**. Biofilm assay with microtiter plates at both 12 hours and 24 hours’ time points showed that YN201732M1 formed a thin and fragile biofilm compared to wild-type strain YN201732, which had no difference with the biofilm of strain YN201732M2 and YN201732M3. The mutants YN201732M1, YN201732M2, and YN201732M3 had an obvious growth lag in the 12h before culture. The biofilm was basically not visible to the naked eye, and the wild-type biofilm was formed. It was found that the biofilm formed by the three mutants, especially YN201732M1, after 24 h of culture was very thin and did not form a spatial three-dimensional structure, and the biomass of the biofilm was very small. However, the biofilm formed by wild-type YN201732 after 24 h of culture had a complex spatial three-dimensional structure, and wrinkles were also observed. It is speculated that lipopeptides may also be a key factor affecting the formation of biofilms.

### Inhibition Effects of *Bacillus amyloliquefaciens* YN201732 and Its Mutants on the Germination of *Erysiphe cichoracearum* Conidia

Comparing the inhibitory effects of wild type and the mutants on the germination of powdery mildew spores (**Table 3**), fermentation filtrates of mutants with different antimicrobial lipopeptides were used to study the effect on the conidia germination. The inhibitory rate of *sfp* gene mutant YN201732M1 on conidial germination was only 30.29%. Antimicrobial lipopeptides may be the main factor that affects spore germination. The spore inhibition rates of *bmyA* gene mutant YN201732M2 and *fenB* gene mutant YN201732M3 were similar (50.22% and 53.06%, respectively), indicating that fengycin and bacillomycin D have significant effects on the spore germination, but YN201732M2 spore germination rate was significantly different from wild type. The difference indicated that bacillomycin D has a major effect on the spore germination of powdery mildew.

### Biocontrol of *Bacillus amyloliquefaciens* YN201732 and Its Mutant Inoculation on Tobacco Powdery Mildew Under Greenhouse Conditions

From the results of YN201732 and its mutants on the management of tobacco powdery mildew (**Table 4**), it can be seen that the mutants have significantly different control effects than the wild type. From days 3 to 30, the control effect of the *sfp* gene mutant YN201732M1 was significantly different from that of wild-type YN201732 and the positive control 50% WG treatment, and there was no significant difference in the negative control. At 7 days, the mutant YN201732M2, YN201732M3 had no significant difference in disease index compared to wild type YN201732 and the positive control, and the control effect was more than 65.46%. Starting from 14 days, compared to the positive control 50% WG, the control effect of other treatments began to decrease. The disease indexes of YN201732M2 and YN201732M3 were significantly lower than the wild-type YN201732 treatment. During days 21 to 30, mutants YN201732M2, YN201732M3, and wild-type YN201732 had no significant difference in disease index, but they were still significantly different from the negative control.

**Table 4:** Control effect of *Bacillus amyloliquefaciens* YN201732 and the mutants on tobacco powdery mildew in the greenhouse.

Treatment	Disease base	3d		7d		14d		21d		30d	
		Disease index	Control effect	Disease index	Control effect	Disease index	Control effect	Disease index	Control effect	Disease index	Control effect
CK	16.84^ab^ ± 2.03	29.02^d^ ± 2.54	–	33.81^c^ ± 2.74	–	42.11^d^ ± 1.14	–	54.14^d^ ± 1.93	–	63.72^d^ ± 2.47	–
YN201732M1	15.41^a^ ± 1.94	18.17^c^ ± 2.22	37.39	25.02^b^ ± 2.09	24.59	35.19^c^ ± 1.87	16.43	46.79^c^ ± 2.33	13.58	59.21^cd^ ± 1.75	7.08
YN201732M2	15.99^a^ ± 0.87	11.14^ab^ ± 1.44	61.61	10.73^a^ ± 0.99	67.66	20.64^b^ ± 1.97	50.99	34.34^b^ ± 2.74	36.57	54.99^bc^ ± 3.08	13.7
YN201732M3	17.41^ab^ ± 2.79	13.10^b^ ± 1.68	54.86	11.46^a^ ± 0.64	65.46	21.32^b^ ± 2.49	49.37	32.59^b^ ± 3.31	39.8	56.07^bc^ ± 3.32	12.01
YN201732	18.81^b^ ± 1.26	9.08^a^ ± 1.10	68.71	9.34^a^ ± 2.75	71.85	14.60^a^ ± 0.98	65.33	29.24^b^ ± 1.59	45.99	52.84^b^ ± 2.59	17.07
Azoxystrobin 50% WG	19.02^b^ ± 3.03	12.76^b^ ± 1.33	56.03	12.58^a^ ± 1.05	62.09	10.68^a^ ± 1.43	74.64	9.17^a^ ± 0.79	83.06	11.85^a^ ± 2.68	81.4

Different letters indicate that the differences are significant (P < 0.05) using Duncan’s multiple range test.

## Discussion

The lipopeptide compounds surfactin, iturin, and fengycin by *B. amyloliquefaciens* are considered to be the main antifungals against plant pathogens (Ongena et al., 2007; Ongena and Jacques, 2008; Li et al., 2019). Grady et al. (2019) purified two antimicrobial lipopeptides (surfactin B and surfactin C) from the fermentation broth of *B. velezensis* strain 9D-6, which inhibited *in vitro* growth of prokaryotic and eukaryotic pathogens. In our study, the plate antagonism test showed that only n-butanol extraction component had potential antagonistic activity against *F. solani*. This component was identified as the lipopeptide bacillomycin D by LCMS-IT-TOF analysis. Lipopeptide bacillomycin D produced by *B. amyloliquefaciens* FZB42 has strong antifungal activity against *F. graminearum*, can cause morphological changes in the plasma membrane and cell wall of *F. graminearum*, and induce reactive oxygen species, further accumulation eventually leads to cell death (Gu et al., 2017). In addition, the main active component of the ethyl acetate extract was detected by mass spectrometry (results not shown), but the effect of surfactin against pathogenic fungi was poor, consistent with previous report (Peypoux et al., 1999). As a biosurfactant, it is related to the limping movement of bacteria and biofilm formation. It has obvious antiviral, antitumor, and antimycobacterial activity, and its antifungal activity is poor. No fengycin compounds were detected in the mass spectrometry results of different solvent-extracted components in this test, but fengycin synthesis-related genes were cloned from the genome. It is speculated that this may be caused by the loss of fengycin during the isolation and purification process. Further research is needed to find underlying reasons.

The results of liquid phase and liquid chromatography-mass spectrometry showed that the compound bacilllomycin D may be the main active substance of bacterial strain YN201732 against *F. solani*. However, due to the complex composition of the fermentation liquid of *Bacillus* and the inclusion of various inorganic and organic compounds, a single isolation and purification method cannot completely separate these large molecular proteins and small molecular compounds. Therefore, the mechanism by which *B. amyloliquefaciens* YN201732 inhibits pathogenic fungi was needed to be further verified by gene knockout. We successfully knocked out three genes related to the synthesis of antimicrobial lipopeptides in endophyte YN201732, the 4’-phosphopantetheinyl transferase gene *sfp*, and the compound bacillomycin D and fengycin synthesis genes *bmyA* and *fenB*. Antagonistic results of the mutants and the indicator fungus *F. solani* showed that the *sfp* gene mutant YN201732M1 lost its antagonism ability, and the pathogenic fungi and strains had almost no inhibitory zones, while the bacillomycin D mutant YN201732M2 antagonism capacity decreased by about 72.44%. Fengycin antagonism was slightly reduced, but it was not significantly different from the wild type. The above results indicate that lipopeptides are the main antagonists that inhibit pathogenic fungi, and bacillomycin D produced by *B. amyloliquefaciens* YN201732 is the main active substance that inhibits the growth of *F. solani*. Previously, both fengycin and bacillomycin D were detected in the culture extract of strain *B. subtilis* 49, with antifungal activities demonstrated against *F. graminearum* and *Sclerotinia sclerotiorum* (Ramarathnam et al., 2007).

Under laboratory conditions, when *B. amyloliquefaciens* grows statically in the culture medium, a biofilm is formed at the interface between the liquid surface and the air (Donlan and Costerton, 2002; Flemming and Wingender, 2010). Pellicle biofilm greatly explains the bacteria’s ability to resist adverse external conditions (Douarche et al., 2015). Three mutants in this study had a certain delay in biofilm formation compared to wild-type strains. The delay in YN201732M1 biofilm formation was the highest, indicating that the 4’-phosphopantetheinyl transferase (*sfp*) not only regulates the synthesis of lipopeptides, it also affects the formation of biofilms. It is known that bacterial biofilm formation ability is closely related to its colonization ability (Xu, 2014), so these active compounds not only kill pathogens by direct antagonizing but also affect their colonization ability through biofilm formation of biocontrol bacteria.

Powdery mildew caused by *E. cichoracearum* seriously affects the yield and quality of tobacco leaves, and once it occurs, it often results in huge economic losses (Glawe, 2008; Meng, 2014). Recent research found that *B. amyloliquefaciens* YN201732 has a good therapeutic and protective effect on tobacco powdery mildew, and the fermentation broth of the strain has a certain inhibitory effect on spore germination (Jiao et al., 2020), but the specific mechanism is still unclear. In this study, bacillomycin D, a lipopeptide compound that inhibits the activity of pathogenic fungi, was isolated and proved to play a major role in antagonizing the indicator pathogen *F. solani*. At the same time, the mutants were used to analyze the effect of the fermentation filtrate on the spore germination. The *sfp* gene mutant YN201732M1 had a poor inhibitory effect on the conidia germination of *E. cichoracearum*. Further analysis found that the spore germination inhibition rate of mutants YN201732M2 and mutant YN201732M3 was reduced, while the germination rate of YN201732M2 spores was significantly different from the wild type, and it was speculated that fengycin and bacillomycin D has an effect on the spore germination, but bacillomycin D has a profound effect. Romero et al. (2007) reported that the iturin and fengycin families of lipopeptides are the key factors for *B. subtilis* to antagonize *Podosphaera fusca* of powdery mildew of Cucurbitaceae, the lipopeptide components had a strong inhibition effect on the conidial germination of *P. fusca.* Greenhouse experiments for mutants also showed that the *sfp* gene YN201732M1 mutant had no control effect, indicating that secretion of lipopeptides is one of the main mechanisms of disease control. The effect of mutant YN201732M2 and mutant YN201732M3 is lower than the wild type and the control effect is equivalent, which means that fengycin and bacillomycin D are the main substances to display inhibition. Bacillomycin D and fengycin antibiotic gene clusters were found by He et al. (2013) in the genome of *B. amyloliquefaciens* B9601-Y2, and MALDI-TOF-MS mass spectrometry detected the cyclic peptide compound bacillomycin D (m (z/z peak at 1031.5-1097.2) and fengycin (m/z peak at 1433.6-1543.2). It was found that bacillomycin D and fengycin are the main antifungal substances, and the two can enhance the antifungal activity of B9601-Y2 through coordination. The results of this study are consistent with previous studies; bacillomycin D and fengycin play an important role in inhibiting the activity of pathogenic fungi. Xu et al. (2013) found for the first time that bacillomycin D can affect the biofilm formation and rhizosphere colonization of *B. amyloliquefaciens* SQR9 and play a major role in antagonizing *F. oxysporum*.

## Conclusions

In this study, crude lipopeptides were obtained from fermentation broth of *B. amyloliquefaciens* YN201732 using acid precipitation and organic solvent extraction methods. Only the n-butanol extracted components had antagonistic activity against indicator pathogen *F. solani*. Through LCMS-IT-TOF analysis, we confirmed that bacillomycin D is the main active substance of endophyte YN201732 against *F. solani*. Combining with the gene knockout method, it was found that the *sfp* gene knockout mutant YN201732M1 inhibited the spore germination and significantly reduced the ability to prevent and control powdery mildew, while the control effects of the *bmyA* gene knockout mutant YN201732M2 and the *fenB* gene knockout mutant YN201732M3 were both lower than the wild type indicating production of antimicrobial lipopeptides by bacteria YN201732 is one of the main mechanism responsible for the management of tobacco powdery mildew. In addition, we also confirmed that mutants of genes related to compounds synthesis also affect biofilm formation.

## Data Availability Statement

The original contributions presented in the study are included in the article/supplementary material. Further inquiries can be directed to the corresponding authors.

## Author Contributions

RJ carried out the conceptualization, data curation, formal analysis, investigation, methodology, funding acquisition, project administration, resource management, supervision, validation, software curation, visualization, and writing (original draft, review, and editing). PFH carried out the conceptualization, data curation, formal analysis, investigation, funding acquisition, project administration, resource management, supervision, validation, software curation, visualization, and writing (original draft, review, and editing). SM carried out the conceptualization, data curation, formal analysis, investigation, methodology, validation, software curation, visualization, and writing (original draft, review, and editing). YW carried out the funding acquisition, project administration, resource management, and supervision. JW carried out the funding acquisition, project administration, resource management, and supervision. MX carried out the conceptualization, data curation, formal analysis, investigation, and methodology. PBH carried out the conceptualization, data curation, formal analysis, investigation, and methodology. HY carried out the conceptualization, resource management, formal analysis, supervision, and methodology. GW carried out the conceptualization, project administration, formal analysis, supervision, and methodology. SK carried out the writing (original draft, review, and editing). YX carried out the funding acquisition, project administration, resource management, supervision, investigation, and methodology. YC carried out the conceptualization, data curation, formal analysis, funding acquisition, investigation, and methodology. YH carried out the conceptualization, data curation, formal analysis, investigation, methodology, funding acquisition, project administration, resource management, supervision, validation, software, visualization, and writing (original draft, review, and editing). All authors contributed to the article and approved the submitted version.

## Funding

This research was financially supported by The National Key Research and Development Program of China (2017YFD0201100), the National Natural Science Foundation of China (Grant no: 31860357), and the Research and Development Foundation of Yunnan Tobacco Company (Grant no: 2019530000241008).

## Conflict of Interest

Authors YX and YC were employed by Qujing Tobacco Co., Ltd. Author JW was employed by the company Hongta Tobacco (Group) Co., Ltd.

The remaining authors declare that the research was conducted in the absence of any commercial or financial relationships that could be construed as a potential conflict of interest.
